# Dietary antioxidants and risk of Parkinson's disease in two population‐based cohorts

**DOI:** 10.1002/mds.27120

**Published:** 2017-09-07

**Authors:** Fei Yang, Alicja Wolk, Niclas Håkansson, Nancy L. Pedersen, Karin Wirdefeldt

**Affiliations:** ^1^ Department of Medical Epidemiology and Biostatistics Karolinska Institutet Stockholm Sweden; ^2^ Institute of Environmental Medicine, Unit of Nutritional Epidemiology, Karolinska Institutet Stockholm Sweden; ^3^ Department of Psychology University of Southern California Los Angeles California USA; ^4^ Department of Clinical Neuroscience Karolinska Institutet Stockholm Sweden

**Keywords:** diet, antioxidants, Parkinson's disease, risk factors

## Abstract

**Background**: A neuroprotective effect of dietary antioxidants on Parkinson's disease (PD) risk has been suggested, but epidemiological evidence is limited.

**Objectives**: To examine the associations between intake of dietary antioxidant vitamins and total antioxidant capacity and risk of PD.

**Methods**: We prospectively assessed the relationships of dietary antioxidant vitamins C and E, ß‐carotene, and total antioxidant capacity with PD risk in two population‐based cohorts (38,937 women and 45,837 men).

**Results**: During a mean 14.9‐year follow‐up period, 1,329 PD cases were identified. Dietary intake of ß‐carotene was associated with a lower risk of PD (hazard ratio: 0.86; 95% confidence interval: 0.78‐0.95; *P*_trend_ < 0.01 for women and hazard ratio: 0.91; 95% confidence interval: 0.84‐0.99; *P*_trend_ = 0.05 for men). An inverse association between dietary vitamin E and PD risk was found in women (hazard ratio: 0.87; 95% confidence interval: 0.79‐0.96; *P*_trend_ = 0.02). Dietary intake of vitamin C was inversely associated with PD risk in women at borderline significance (hazard ratio: 0.91; 95% confidence interval: 0.83‐1.00; *P*_trend_ = 0.04). There was no association between dietary total antioxidant capacity and PD risk in either women (hazard ratio: 0.93; 95% confidence interval: 0.84‐1.02; *P*_trend_ = 0.35) or men (hazard ratio: 1.00; 95% confidence interval: 0.93‐1.07; *P*_trend_ = 0.97).

**Conclusion**: Intake of dietary vitamin E and ß‐carotene was associated with a lower risk of PD. © 2017 The Authors. Movement Disorders published by Wiley Periodicals, Inc. on behalf of International Parkinson and Movement Disorder Society.

Dietary antioxidants including vitamin C, E, and carotenoids have been suggested as neuroprotective agents for Parkinson's disease (PD) based on their property of reducing oxidative damage.^1^ Epidemiological evidence for a neuroprotective effect of dietary antioxidants on PD risk is, however, largely limited and inconsistent.^2^, ^3^ In addition, although not only vitamin C, E, and carotenes, but also several other compounds are dietary antioxidants, no previous study has yet examined the role of total dietary antioxidants on PD risk.

In this study, we estimated total antioxidant capacity (TAC) in a single estimate by taking into account summed effects of compounds from all relevant dietary antioxidants in the foods. We prospectively investigated the relationship of TAC, as well as the individual dietary antioxidant vitamins C and E and ß‐carotene, with PD risk in two population‐based cohorts.

## Materials and Methods

### Study Population

We used data from the Swedish Mammography Cohort (SMC) and the Cohort of Swedish Men (COSM). In brief, the SMC was created to investigate the relationship of dietary and hormonal factors with breast cancer risk in all women born between 1914 and 1948 and living in the Västmanland and Uppsala counties in central Sweden. Those who were still alive and living in the area were contacted and completed a 350‐item mailed questionnaire asking about lifestyle and other risk factors for noncommunicable diseases during the late autumn of 1997 (n = 38,984; 70% response rate). The COSM consists of all men who were born between 1918 and 1952 and lived in the Västmanland and Örebro counties in 1997. They answered a questionnaire identical to the one used in SMC, except for some sex‐specific questions (n = 45,906; 49% response rate).

After exclusion of prevalent PD cases (n = 116), in total 84,774 participants (38,937 women and 45,837 men) who were alive at start of follow‐up were included in this study. Baseline was 15 September 1997 for the SMC and 1 January 1998 for the COSM. The present study was approved by the Regional Ethics Review Board in Stockholm, Sweden.

### Assessment of Dietary Antioxidants, TAC, and Covariates

In both SMC and COSM baseline questionnaires, a 96‐item food frequency questionnaire (FFQ) tailored to the Swedish diet was used to assess the participants' dietary habits. The FFQ was a part of the 350‐item mailed questionnaire. Participants were required to report their exact consumption per day or per week for each type of food component during the past year.

Intake of dietary antioxidant vitamins C and E and ß‐carotene was calculated by multiplying the mean frequency of each food item by the nutrient content of age‐ and sex‐specific portion sizes, using food composition values from the Swedish Food Administration Database.^4^ Similarly, we calculated dietary TAC by multiplying the mean frequency of each food item by the oxygen radical absorbance capacity (ORAC) value (μmol Trolox equivalent/100 g) of age‐ and sex‐specific portion sizes, by linkage to a database of the most common foods analyzed with the ORAC assay.^5^, ^6^, ^7^ The TAC calculation has been described in detail elsewhere.^8^ Intake of dietary antioxidant vitamins and TAC were all adjusted for total energy intake with the residual method,^9^ and dietary TAC estimate was further adjusted for poor absorption of antioxidants from coffee and tea consumption.^10^ The FFQs have previously been described and validated, showing a mean correlation coefficient of 0.62 between self‐reported micronutrient estimates and 24‐hour recall interviews^11^, ^12^ and a correlation coefficient of 0.31 between self‐reported dietary TAC and plasma ORAC values.^8^

### PD Case Ascertainment

Both prevalent and incident PD cases were identified by linkage with the Swedish National Patient Register and the Cause of Death Register to obtain first‐ever primary or secondary inpatient and outpatient PD diagnoses, as well as death records with PD as the underlying or contributing cause of death. Both cohorts were completely linked to these national registers. The Swedish revision of the International Classification of Diseases (ICD) codes were used for PD diagnoses: 350 (ICD‐7, 1964‐68), 342 (ICD‐8, 1969‐86), 332.0 (ICD‐9, 1987‐96), and G20 (ICD‐10, 1997‐2014). A previous validation study reported a positive predictive value of 70.8% and a sensitivity of 72.7%, comparing hospital discharge diagnoses of PD in the registers against clinical diagnoses.^13^

### Statistical Analysis

All participants were followed from baseline until date of PD diagnosis, death, or end of follow‐up (31 December 2014), whichever came first. A Cox proportional hazard regression model with attained age as underlying time scale was used to estimate relative risks as hazard ratios (HRs) with 95% confidence intervals (CIs). We stratified the analyses by sex. Intake of dietary antioxidant vitamins and TAC were first categorized into quartiles and analyzed with the lowest category as the reference group. Trend analyses were conducted using the median of each exposure category as a single ordinal variable in the model. Then, we performed analyses for the intake of dietary antioxidant vitamins and TAC as continuous variables, using an approximate value of 1 standard deviation (SD) of the mean dietary intake in the study population as per unit.

We adjusted for potential confounders in the multivariable model, including smoking status (never, former, or current smokers), intake of alcohol from all types of alcoholic beverage (ethanol, g/day) and coffee (g/day), body mass index (BMI; low, < 18.5 kg/m^2^; normal, 18.5‐24.9 kg/m^2^; overweight, 25.0‐29.9 kg/m^2^; and obese: ≥ 30 kg/m^2^), highest achieved level of education (compulsory school, secondary or high school, and university or above), multivitamin supplement use (never/sometimes/regular), and total energy intake (kcal/day). To address the possibility of preclinical PD at baseline, we also performed lag‐time analyses by excluding the first 4 and then the first 8 years of follow up. Given that smoking is the most consistent variable related to PD risk, to better control for potential residual confounding by smoking, we further conducted sensitivity analyses stratified by smoking status. All statistical analyses were performed in R software (version 3.2.3; R Foundation for Statistical Computing, Vienna, Austria).

## Results

Participants' baseline characteristics are presented in Table 1. Individuals with higher intake of dietary antioxidant vitamins and TAC were less likely to be current smokers, but more likely to have ≥12 years of education and to use multivitamin supplements regularly. On average, men had higher vitamin E intake, dietary TAC and total energy intake, and drank more alcohol and coffee than women. During a mean 14.9‐year (SD, 4.1) follow‐up period, 1,329 incident PD cases were identified (518 women and 811 men). Incidence of PD increased with age and was lower for smokers than nonsmokers for both women (incidence rate ratio [IRR]: 0.52; 95% CI: 0.43‐0.63) and men (IRR, 0.68; 95% CI: 0.59‐0.79). Mean age at PD diagnosis was 75.7 years (SD, 7.8) for women and 74.6 years (SD, 7.8) for men.

**Table 1:** Characteristics of participants in SMC and COSM at baseline in 1997

	Vitamin C		Vitamin E		ß‐carotene		TAC of diet		
	First Quartile	Fourth Quartile	First Quartile	Fourth Quartile	First Quartile	Fourth Quartile	First Quartile	Fourth Quartile	Overall
Women (n = 38,937)									
Age (mean, years)	63.9	60.9	64.0	61.3	61.9	63.3	62.9	61.7	62.2
Intake of dietary antioxidants^a^									
Vitamin C (mg/day)	55.6 ± 15.4	188.6 ± 49.5	81.7 ± 38.3	151.5 ± 68.5	79.6 ± 40.6	150.6 ± 64.8	72.4 ± 31.5	167.1 ± 63.0	113.6 ± 56.5
Vitamin E (mg/day)	6.0 ± 1.1	7.5 ± 1.2	5.3 ± 0.5	8.2 ± 0.8	6.0 ± 1.1	7.4 ± 1.2	6.0 ± 1.2	7.4 ± 1.1	6.7 ± 1.2
ß‐carotene (mg/day)	2.2 ± 1.5	4.7 ± 2.6	2.4 ± 1.5	4.6 ± 2.6	1.4 ± 0.5	6.2 ± 2.1	2.3 ± 1.5	4.6 ± 2.6	3.4 ± 2.1
TAC (TE/day)	9,863 ± 2,511	16,514 ± 4,152	10,531 ± 2,940	15,290 ± 4,611	10,696 ± 3,301	15,130 ± 4,165	8,461 ± 1,365	18,063 ± 3,067	12,840 ± 3,932
Total energy intake (kcal/day)	1,748	1,690	1,745	1,729	1,740	1,678	1,753	1,722	1,727
Alcohol (ethanol, mean, g/day)	3.5	4.3	4.1	3.7	4.1	3.5	3.6	4.2	4.1
Coffee (mean, g/day)	586.6	504.0	570.1	515.2	582.8	505.8	457.7	608.3	541.5
BMI (mean, kg/m^2^)	25.0	25.1	25.0	25.2	24.9	25.2	25.1	25.1	25.0
Smoking status (%)									
Never	52.5	53.1	52.3	53.6	50.1	56.7	50.9	55.5	53.9
Former	19.6	26.3	20.1	25.3	21.5	23.8	21.7	24.8	22.9
Current	28.0	20.7	27.7	21.1	28.4	19.5	27.4	19.7	23.2
Education level (%)									
Compulsory	54.0	32.9	49.8	37.0	47.6	40.6	48.6	36.3	42.2
High school	34.8	42.7	36.6	41.7	37.1	41.2	37.8	41.4	39.5
University and above	11.2	24.4	13.6	21.3	15.3	18.2	13.6	22.3	18.3
Multivitamin supplement use (%)									
Never	48.9	43.8	49.2	43.8	50.5	42.5	48.6	44.0	46.1
Sometimes	28.2	27.8	27.1	28.2	27.5	28.2	28.8	27.8	29.0
Regular	22.9	28.4	23.7	28.0	22.0	29.3	22.6	28.3	24.9
Men (n = 45,837)									
Age (mean, y)	61.7	60.5	62.8	60.1	60.9	61.7	60.6	61.6	60.8
Intake of dietary antioxidants^a^									
Vitamin C (mg/day)	50.2 ± 14.2	184.0 ± 49.9	88.8 ± 48.5	132.3 ± 67.9	77.6 ± 44.6	140.8 ± 64.6	78.1 ± 40.5	151.4 ± 66.6	108.5 ± 56.3
Vitamin E (mg/day)	8.3 ± 1.6	9.4 ± 1.7	7.0 ± 0.7	10.9 ± 1.1	8.1 ± 1.6	9.5 ± 1.5	8.1 ± 1.6	9.6 ± 1.5	8.8 ± 1.6
ß‐carotene (mg/day)	1.8 ± 1.3	3.8 ± 2.3	2.0 ± 1.4	3.6 ± 2.3	1.1 ± 0.4	5.3 ± 1.9	2.0 ± 1.3	3.8 ± 2.3	2.8 ± 1.8
TAC (TE/day)	11,901 ± 3,054	17,549 ± 4,799	12,221 ± 3,445	16,479 ± 4,817	12,376 ± 3,707	16,675 ± 4,515	9,692 ± 1,552	20,047 ± 3,237	14,471 ± 4,225
Total energy intake (kcal/day)	2,674	2,626	2,672	2,632	2,657	2,595	2,667	2,611	2,648
Alcohol (mean, g/dad)	10.3	10.3	11.6	9.1	10.1	9.7	10.8	9.9	10.3
Coffee (mean, g/day)	782.1	639.0	732.9	686.4	762.6	652.7	557.4	817.1	706.9
BMI (mean, kg/m^2^)	25.7	25.9	25.9	25.8	25.9	25.7	26.0	25.7	25.8
Smoking status (%)									
Never	30.5	39.9	31.3	38.1	31.2	40.2	31.8	39.8	36.2
Former	37.6	40.1	38.4	39.8	37.2	39.8	38.3	39.8	38.9
Current	31.9	20.0	30.3	22.1	31.5	20.0	30.0	20.4	25.0
Education level (%)									
Compulsory	44.1	27.5	43.7	30.9	42.6	31.5	39.6	31.1	35.0
High school	45.4	50.1	45.4	50.0	46.0	49.1	48.3	49.5	48.8
University and above	10.5	22.4	10.9	19.2	11.5	19.4	12.0	19.4	16.2
Multivitamin supplement use (%)									
Never	72.4	62.8	72.0	64.3	73.4	62.1	72.6	62.6	67.8
Sometimes	15.1	17.8	15.1	18.4	14.4	18.6	15.4	17.8	17.3
Regular	12.5	19.4	12.9	17.3	12.2	19.3	12.0	19.6	14.9

TAC is an index score measured in micromole Trolox equivalents (TE) with the oxygen radical capacity absorbance assay, based on individual answers from FFQ.

Percentages may not add up to 100% because of rounding.

a Age‐standardized values are presented in means ± SD and based on the first and last quartiles of intake of dietary antioxidants.

Dietary intake of ß‐carotene was associated with a lower risk of PD in both women and men (Table 2). There was an inverse association between dietary intake of vitamin E and PD risk in women, but the inverse association was only observed in men when vitamin E intake was analyzed as a continuous variable. Dietary vitamin C intake was inversely associated with PD risk in women at borderline significance. There was no association between dietary TAC and PD risk.

**Table 2:** Associations between daily intake of dietary antioxidants and risk of PD

	SMC (Women)					COSM (Men)			
	n	HR^a^	95% CI^a^	*P* Value^a^		n	HR^a^	95% CI^a^	*P* Value^a^
Vitamin C					Vitamin C				
Quartiles^b^					Quartiles^b^				
<74.3 (57.4)	104	1	Ref		<69.8 (52.5)	129	1	Ref	
74.3‐103.9 (88.9)	113	1.00	0.77—1.29		69.8‐98.6 (84.3)	153	1.09	0.87—1.37	
103.9‐141.9 (120.8)	97	0.86	0.65—1.12		98.6‐136.0 (115.3)	170	1.19	0.95—1.49	
>141.9 (174.4)	82	0.77	0.58—1.03		>136.0 (169.5)	170	1.15	0.92—1.45	
Trend				0.04	Trend				0.23
Per 50 mg/day	396	0.91	0.83—1.00	0.05	Per 50 mg/day	622	1.02	0.95—1.09	0.60
Vitamin E					Vitamin E				
Quartiles^b^					Quartiles^b^				
<5.9 (5.4)	122	1	Ref		<7.8 (7.1)	148	1	Ref	
5.9‐6.6 (6.3)	89	0.71	0.54—0.92		7.8‐8.7 (8.3)	160	0.99	0.79—1.22	
6.6‐7.4 (7.0)	102	0.82	0.64—1.06		8.7‐9.7 (9.2)	176	1.05	0.85—1.30	
>7.4 (8.0)	83	0.69	0.52—0.90		>9.7 (10.5)	138	0.85	0.68—1.07	
Trend				0.02	Trend				0.23
Per 1.2 mg/day	396	0.87	0.79—0.96	<0.01	Per 1.2 mg/day	622	0.93	0.88—0.99	0.02
ß‐carotene					ß‐carotene				
Quartiles^b^					Quartiles^b^				
<1.9 (1.4)	108	1	Ref		<1.6 (1.1)	158	1	Ref	
1.9‐2.9 (2.4)	97	0.82	0.63—1.07		1.6‐2.3 (1.9)	154	0.85	0.69—1.06	
2.9‐4.4 (3.6)	97	0.77	0.59—1.00		2.3‐3.6 (2.8)	152	0.79	0.64—0.99	
>4.4 (5.6)	94	0.69	0.53—0.91		>3.6 (4.8)	158	0.79	0.63—0.98	
Trend				0.01	Trend				0.05
Per 2 mg/day	396	0.86	0.78—0.95	<0.01	Per 2 mg/day	622	0.91	0.84—0.99	0.03
TAC					TAC				
Quartiles^c^					Quartiles^c^				
<10,173 (8,772)	94	1	Ref		<11,614 (10,039)	132	1	Ref	
10,173‐12,353 (11,283)	105	0.95	0.73—1.24		11,614‐14,025 (12,841)	144	0.89	0.70—1.12	
12,353‐14,966 (13,507)	93	0.83	0.63—1.10		14,025‐16,789 (15,249)	164	0.94	0.75—1.17	
>14,967 (17,175)	104	0.89	0.68—1.18		>16,789 (19,102)	182	0.96	0.77—1.21	
Trend				0.35	Trend				0.97
Per 4,000 TE/day	396	0.93	0.84—1.02	0.13	Per 4,000 TE/day	622	1.00	0.93—1.07	0.92

n = number of PD cases in the analyses. TAC is an index score measured in micromole Trolox equivalents (TE) with the oxygen radical capacity absorbance assay, based on individual answers from FFQ.

a Cox model with attained‐age as time scale, adjusting for smoking (never/former/current), intake of alcohol (ethanol, g/day, continuous) and coffee (g/day, continuous), education (compulsory/high school/university), BMI (<18.5 kg/m^2^, 18.5‐24.9 kg/m^2^, 25‐29.9 kg/m^2^, ≥ 30 kg/m^2^), total energy intake (kcal/day, continuous), and multivitamin supplement use (never/sometimes/regular).

b Values are presented with range in mg/day (median).

c Values are presented with range in TE/day (median).

In the 4‐year lagged sensitivity analysis, the inverse association remained significant for dietary intake of ß‐carotene in both women and men and for vitamin E intake in women (Supplementary Table 1). Our 8‐year lagged analysis showed comparable results (Supplementary Table 2). Results were similar in the sensitivity analyses stratified by smoking (Supplementary Tables 3 and 4).

## Discussion

Our results showed that dietary intake of ß‐carotene was associated with a lower risk of PD. An inverse association between dietary intake of vitamin E and PD risk was found in women, but not in men. Dietary vitamin C intake was inversely associated with PD risk in women at borderline significance, but not in men. We did not observe an association between TAC and PD risk.

Few studies examined the associations between intake of dietary vitamins C, E, and ß‐carotene and PD risk, with inconsistent findings from either case‐control^14^, ^15^, ^16^, ^17^, ^18^ or prospective cohort designs.^19^, ^20^, ^21^, ^22^ The inverse association between dietary vitamin E intake and PD risk in women in our study is in line with a previous prospective study showing that intake of dietary vitamin E (from foods only), but not intake of total vitamin E (from both foods and supplements), was associated with reduced PD risk.^23^ Generally, women have a lower risk of PD than men and antioxidant activity of estrogens has been suggested to protect against PD.^24^ Thus, in our study, the observed inverse association for dietary intake of vitamin E intake in women, but not in men, might be attributable to an effect modification of estrogens. However, a meta‐analysis of observational studies reported that both moderate and high intake of dietary vitamin E were associated with lower risk of PD.^25^ In addition, the same meta‐analysis reported no protective effect associated with vitamin C or ß‐carotene, whereas we found that dietary ß‐carotene intake was associated with lower PD risk in both women and men. Interestingly, another recent meta‐analysis reported an inverse, but nonsignificant, association between dietary intake of ß‐carotene and PD risk.^26^

To the best of our knowledge, this is the first prospective study examining the association between dietary TAC and PD risk. The null finding between dietary TAC and PD risk might be attributed to a relatively low correlation between self‐reported dietary TAC and plasma ORAC values. Another possible explanation could be that some dietary antioxidant vitamins (such as vitamin C) do not affect the risk of PD, resulting in an attenuated association between dietary TAC and PD risk when including vitamin C in the TAC estimate. A possible reason why vitamin C may not affect the risk of PD despite its antioxidant activity might be that it is water soluble and does not easily cross the blood–brain barrier.^27^

The mechanisms of dopaminergic neuron death in PD and the roles of vitamin E and ß‐carotene in the central nervous system have not yet been fully elucidated. However, a protective effect of vitamin E and ß‐carotene on PD risk is biologically plausible through reducing oxidative damage by neutralizing the effect of oxygen free radicals, as shown from in vitro and in vivo studies.^28^, ^29^, ^30^, ^31^ For example, our observed inverse association between dietary intake of vitamin E and PD risk could be explained by modulation of expression of the MAPT gene by vitamin E.^32^ Thus, all evidence together indicates that foods rich in vitamin E and ß‐carotene may protect against PD.

Strengths of our study include the population‐based prospective design with long follow‐up, large sample size of both sexes, use of validated nutrient measurements, and records of PD diagnosis in nationwide health registers, limiting the risks of recall bias and reverse causation. Nevertheless, our study has some limitations. Misclassification of dietary intake of antioxidant vitamins is possible, although the FFQs were validated and tailored to the Swedish diet and estimates on dietary intake from the FFQs were linked to a standard Swedish Food Administration database.^11^, ^12^ However, in a prospective study design, such misclassification would be nondifferential, leading to bias toward the null and the true association would be stronger than the observed. The use of health registers to identify PD cases is also a limitation. A previous validation study that compared PD register diagnoses against clinical diagnoses showed that although specificity was almost perfect, misclassification between PD and other parkinsonism was common.^13^ However, such misclassification would be nondifferential by exposure and theoretically lead to attenuation of the association between dietary intake of antioxidants and PD risk. It is possible that preclinical PD occurred earlier than the baseline dietary measurement and altered participants' dietary habits. For example, prodromal PD might increase consumption of fiber (for constipation), which could have masked a protective effect of vitamin C on PD risk. However, our lag‐time analyses showed comparable results, suggesting that reverse causation is unlikely. Another limitation is the sex difference in response rate to the questionnaire. Other factors associated with nonresponse in men might also be related to diet and PD, but it is unclear in what direction such factors might bias our associations. It is also possible that a low correlation between TAC and plasma ORAC prevented detection of an association, even when an association exists. Therefore, a better measurement of TAC is warranted in future studies.

In conclusion, our results from two large, population‐based, prospective cohorts suggest that intake of dietary vitamin E and ß‐carotene was associated with a lower risk of PD.

## Author Roles

(1) Research Project: A. Conception, B. Organization, C. Execution; (2) Statistical Analysis: A. Design, B. Execution, C. Review and Critique; (3) Manuscript: A. Writing of the First Draft, B. Review and Critique.

F.Y.: 1A, 1B, 1C, 2A, 2B, 2C, 3A, 3B

A.W.: 1A, 1B, 2C, 3B

N.H.: 1A, 1B, 3B

N.L.P.: 1A, 1B, 1C, 2C, 3B

K.W.: 1A, 1B, 1C, 2C, 3B

## Financial Disclosures

A.W. has ongoing grants from the Swedish Cancer Society, FORTE, FORMAS, and EU‐Horizon 2020.

## Supporting information

Additional Supporting Information may be found in the online version of this article

Supplementary Information Tables.
