# Supplementary material for: Dietary antioxidants and risk of Parkinson's disease in two population‐based cohorts
Source: Mov Disord. 2017 Sep 7;32(11):1631–6. doi: 10.1002/mds.27120 (PMC5698752; doi:10.1002/mds.27120)
Supplement: Supplementary file 1 — Supplementary Information Tables. [file MDS-32-1631-s001.docx]

**Supplementary Table 1. Associations between daily intake of dietary antioxidants and risk of Parkinson’s disease (4-year lagged analysis)**

|  |  | |  |  |  |  |  | |  |  |  |  |  |  |
| --- | --- | --- | --- | --- | --- | --- | --- | --- | --- | --- | --- | --- | --- | --- |
|  |  | | SMC (women) | | | | | |  | COSM (men) | | | | |
|  |  | | HR^a^ | 95% CI^a^ | | | | *P*-value^a^ |  | HR^a^ | 95% CI^a^ | | | *P*-value^a^ |
| Vitamin C | | |  |  |  |  | |  |  |  |  |  |  |  |
|  | Quartiles | |  |  |  |  | |  |  |  |  |  |  |  |
|  |  | Q1 | 1 | Ref | | | |  |  | 1 | Ref | | |  |
|  |  | Q2 | 1.06 | 0.80 | - | 1.38 | |  |  | 1.15 | 0.90 | - | 1.47 |  |
|  |  | Q3 | 0.94 | 0.71 | - | 1.24 | |  |  | 1.20 | 0.94 | - | 1.53 |  |
|  |  | Q4 | 0.83 | 0.62 | - | 1.12 | |  |  | 1.16 | 0.90 | - | 1.49 |  |
|  | Trend | |  |  |  |  | | 0.14 |  |  |  |  |  | 0.32 |
|  | Per 50 mg/day | | 0.93 | 0.84 | - | 1.02 | | 0.10 |  | 1.01 | 0.94 | - | 1.09 | 0.72 |
|  |  | |  |  |  |  | |  |  |  |  |  |  |  |
| Vitamin E | | |  |  |  |  | |  |  |  |  |  |  |  |
|  | Quartiles | |  |  |  |  | |  |  |  |  |  |  |  |
|  |  | Q1 | 1 | Ref | | | |  |  | 1 | Ref | | |  |
|  |  | Q2 | 0.70 | 0.53 | - | 0.92 | |  |  | 0.99 | 0.78 | - | 1.25 |  |
|  |  | Q3 | 0.78 | 0.60 | - | 1.02 | |  |  | 1.05 | 0.83 | - | 1.33 |  |
|  |  | Q4 | 0.66 | 0.50 | - | 0.88 | |  |  | 0.88 | 0.68 | - | 1.12 |  |
|  | Trend | |  |  |  |  | | 0.01 |  |  |  |  |  | 0.34 |
|  | Per 1.2 mg/day | | 0.86 | 0.77 | - | 0.95 | | 0.00 |  | 0.94 | 0.88 | - | 1.00 | 0.06 |
|  |  | |  |  |  |  | |  |  |  |  |  |  |  |
| ß-carotene | | |  |  |  |  | |  |  |  |  |  |  |  |
|  | Quartiles | |  |  |  |  | |  |  |  |  |  |  |  |
|  |  | Q1 | 1 | Ref | | | |  |  | 1 | Ref | | |  |
|  |  | Q2 | 0.82 | 0.62 | - | 1.08 | |  |  | 0.90 | 0.71 | - | 1.13 |  |
|  |  | Q3 | 0.79 | 0.60 | - | 1.04 | |  |  | 0.77 | 0.61 | - | 0.98 |  |
|  |  | Q4 | 0.71 | 0.53 | - | 0.94 | |  |  | 0.83 | 0.66 | - | 1.05 |  |
|  | Trend | |  |  |  |  | | 0.02 |  |  |  |  |  | 0.14 |
|  | Per 2 mg/day | | 0.87 | 0.79 | - | 0.96 | | 0.01 |  | 0.92 | 0.83 | - | 1.00 | 0.06 |
|  |  | |  |  |  |  | |  |  |  |  |  |  |  |
| TAC of the diet | | |  |  |  |  | |  |  |  |  |  |  |  |
|  | Quartiles | |  |  |  |  | |  |  |  |  |  |  |  |
|  |  | Q1 | 1 | Ref | | | |  |  | 1 | Ref | | |  |
|  |  | Q2 | 0.92 | 0.70 | - | 1.22 | |  |  | 0.96 | 0.75 | - | 1.23 |  |
|  |  | Q3 | 0.83 | 0.63 | - | 1.11 | |  |  | 0.95 | 0.74 | - | 1.22 |  |
|  |  | Q4 | 0.91 | 0.69 | - | 1.21 | |  |  | 0.99 | 0.78 | - | 1.27 |  |
|  | Trend | |  |  |  |  | | 0.51 |  |  |  |  |  | 0.99 |
|  | Per 4000 TE/day | | 0.95 | 0.86 | - | 1.05 | | 0.32 |  | 1.00 | 0.92 | - | 1.08 | 0.92 |
|  |  | |  |  |  |  | |  |  |  |  |  |  |  |

SMC = Swedish Mammography Cohort; COSM = Cohort of Swedish Men; HR = hazard ratio; CI = confidence interval; TAC = Total antioxidant capacity, an index score measured in micromole Trolox equivalents (TE) with the oxygen radical capacity absorbance assay, based on individual answers from FFQ.

^a^ Cox model with attained-age as timescale, adjusting for smoking (never/former/current), intake of alcohol (ethanol, g/day, continuous) and coffee (g/day, continuous), education (compulsory/high school/university), body mass index (<18.5 kg/m^2^, 18.5-24.9 kg/m^2^, 25-29.9 kg/m^2^, ≥30 kg/m^2^), total energy intake (kcal/day, continuous), and multivitamin supplement use (never/sometimes/regular).

**Supplementary Table 2. Associations between daily intake of dietary antioxidants and risk of Parkinson’s disease (8-year lagged analysis)**

|  |  | |  |  |  |  |  |  |  |  |  |  |  |
| --- | --- | --- | --- | --- | --- | --- | --- | --- | --- | --- | --- | --- | --- |
|  |  | | SMC (women) | | | | |  | COSM (men) | | | | |
|  |  | | HR^a^ | 95% CI^a^ | | | *P*-value^a^ |  | HR^a^ | 95% CI^a^ | | | *P*-value^a^ |
| Vitamin C | | |  |  |  |  |  |  |  |  |  |  |  |
|  | Quartiles | |  |  |  |  |  |  |  |  |  |  |  |
|  |  | Q1 | 1 | Ref | | |  |  | 1 | Ref | | |  |
|  |  | Q2 | 0.99 | 0.72 | - | 1.38 |  |  | 1.05 | 0.78 | - | 1.42 |  |
|  |  | Q3 | 0.91 | 0.65 | - | 1.28 |  |  | 1.16 | 0.86 | - | 1.55 |  |
|  |  | Q4 | 0.78 | 0.54 | - | 1.11 |  |  | 1.07 | 0.79 | - | 1.44 |  |
|  | Trend | |  |  |  |  | 0.13 |  |  |  |  |  | 0.66 |
|  | Per 50 mg/day | | 0.94 | 0.84 | - | 1.05 | 0.25 |  | 1.00 | 0.92 | - | 1.10 | 0.93 |
|  |  | |  |  |  |  |  |  |  |  |  |  |  |
| Vitamin E | | |  |  |  |  |  |  |  |  |  |  |  |
|  | Quartiles | |  |  |  |  |  |  |  |  |  |  |  |
|  |  | Q1 | 1 | Ref | | |  |  | 1 | Ref | | |  |
|  |  | Q2 | 0.71 | 0.51 | - | 1.00 |  |  | 0.93 | 0.69 | - | 1.26 |  |
|  |  | Q3 | 0.82 | 0.60 | - | 1.14 |  |  | 1.22 | 0.92 | - | 1.62 |  |
|  |  | Q4 | 0.70 | 0.50 | - | 0.98 |  |  | 1.04 | 0.78 | - | 1.40 |  |
|  | Trend | |  |  |  |  | 0.08 |  |  |  |  |  | 0.48 |
|  | Per 1.2 mg/day | | 0.86 | 0.76 | - | 0.98 | 0.02 |  | 1.00 | 0.92 | - | 1.08 | 0.90 |
|  |  | |  |  |  |  |  |  |  |  |  |  |  |
| ß-carotene | | |  |  |  |  |  |  |  |  |  |  |  |
|  | Quartiles | |  |  |  |  |  |  |  |  |  |  |  |
|  |  | Q1 | 1 | Ref | | |  |  | 1 | Ref | | |  |
|  |  | Q2 | 0.85 | 0.61 | - | 1.19 |  |  | 0.84 | 0.64 | - | 1.11 |  |
|  |  | Q3 | 0.77 | 0.55 | - | 1.08 |  |  | 0.74 | 0.56 | - | 0.98 |  |
|  |  | Q4 | 0.82 | 0.58 | - | 1.14 |  |  | 0.79 | 0.60 | - | 1.05 |  |
|  | Trend | |  |  |  |  | 0.27 |  |  |  |  |  | 0.15 |
|  | Per 2 mg/day | | 0.91 | 0.81 | - | 1.03 | 0.13 |  | 0.93 | 0.83 | - | 1.04 | 0.18 |
|  |  | |  |  |  |  |  |  |  |  |  |  |  |
| TAC of the diet | | |  |  |  |  |  |  |  |  |  |  |  |
|  | Quartiles | |  |  |  |  |  |  |  |  |  |  |  |
|  |  | Q1 | 1 | Ref | | |  |  | 1 | Ref | | |  |
|  |  | Q2 | 0.95 | 0.68 | - | 1.34 |  |  | 0.95 | 0.71 | - | 1.27 |  |
|  |  | Q3 | 0.86 | 0.61 | - | 1.22 |  |  | 0.87 | 0.65 | - | 1.18 |  |
|  |  | Q4 | 0.88 | 0.62 | - | 1.24 |  |  | 0.93 | 0.69 | - | 1.25 |  |
|  | Trend | |  |  |  |  | 0.41 |  |  |  |  |  | 0.63 |
|  | Per 4000 TE/day | | 0.93 | 0.82 | - | 1.05 | 0.24 |  | 1.00 | 0.91 | - | 1.10 | 0.95 |
|  |  | |  |  |  |  |  |  |  |  |  |  |  |

SMC = Swedish Mammography Cohort; COSM = Cohort of Swedish Men; HR = hazard ratio; CI = confidence interval; TAC = Total antioxidant capacity, an index score measured in micromole Trolox equivalents (TE) with the oxygen radical capacity absorbance assay, based on individual answers from FFQ.

^a^ Cox model with attained-age as timescale, adjusting for smoking (never/former/current), intake of alcohol (ethanol, g/day, continuous) and coffee (g/day, continuous), education (compulsory/high school/university), body mass index (<18.5 kg/m^2^, 18.5-24.9 kg/m^2^, 25-29.9 kg/m^2^, ≥30 kg/m^2^), total energy intake (kcal/day, continuous), and multivitamin supplement use (never/sometimes/regular).

**Supplementary Table 3. Associations between daily intake of dietary antioxidants and risk of Parkinson’s disease among never-smokers**

|  |  | |  | | | | |  |  | | | | |
| --- | --- | --- | --- | --- | --- | --- | --- | --- | --- | --- | --- | --- | --- |
|  |  | | SMC (women) | | | | |  | COSM (men) | | | | |
|  |  | | HR^a^ | 95% CI^a^ | | | *P*-value^a^ |  | HR^a^ | 95% CI^a^ | | | *P*-value^a^ |
| Vitamin C | | |  |  |  |  |  |  |  |  |  |  |  |
|  | Quartiles | |  |  |  |  |  |  |  |  |  |  |  |
|  |  | Q1 | 1 | Ref | | |  |  | 1 | Ref | | |  |
|  |  | Q2 | 0.98 | 0.71 | - | 1.35 |  |  | 0.92 | 0.64 | - | 1.32 |  |
|  |  | Q3 | 0.93 | 0.67 | - | 1.29 |  |  | 1.08 | 0.77 | - | 1.53 |  |
|  |  | Q4 | 0.86 | 0.61 | - | 1.21 |  |  | 1.16 | 0.83 | - | 1.63 |  |
|  | Trend | |  |  |  |  | 0.35 |  |  |  |  |  | 0.24 |
|  | Per 50 mg/day | | 0.94 | 0.84 | - | 1.05 | 0.30 |  | 1.02 | 0.93 | - | 1.13 | 0.67 |
|  |  | |  |  |  |  |  |  |  |  |  |  |  |
| Vitamin E | | |  |  |  |  |  |  |  |  |  |  |  |
|  | Quartiles | |  |  |  |  |  |  |  |  |  |  |  |
|  |  | Q1 | 1 | Ref | | |  |  | 1 | Ref | | |  |
|  |  | Q2 | 0.68 | 0.49 | - | 0.93 |  |  | 0.91 | 0.66 | - | 1.27 |  |
|  |  | Q3 | 0.81 | 0.59 | - | 1.09 |  |  | 0.95 | 0.69 | - | 1.32 |  |
|  |  | Q4 | 0.65 | 0.46 | - | 0.90 |  |  | 0.83 | 0.59 | - | 1.17 |  |
|  | Trend | |  |  |  |  | 0.02 |  |  |  |  |  | 0.40 |
|  | Per 1.2 mg/day | | 0.85 | 0.75 | - | 0.96 | 0.01 |  | 0.92 | 0.83 | - | 1.01 | 0.07 |
|  |  | |  |  |  |  |  |  |  |  |  |  |  |
| ß-carotene | | |  |  |  |  |  |  |  |  |  |  |  |
|  | Quartiles | |  |  |  |  |  |  |  |  |  |  |  |
|  |  | Q1 | 1 | Ref | | |  |  | 1 | Ref | | |  |
|  |  | Q2 | 0.98 | 0.71 | - | 1.36 |  |  | 0.78 | 0.56 | - | 1.09 |  |
|  |  | Q3 | 0.91 | 0.66 | - | 1.27 |  |  | 0.77 | 0.56 | - | 1.07 |  |
|  |  | Q4 | 0.82 | 0.58 | - | 1.14 |  |  | 0.73 | 0.53 | - | 1.02 |  |
|  | Trend | |  |  |  |  | 0.19 |  |  |  |  |  | 0.18 |
|  | Per 2 mg/day | | 0.90 | 0.80 | - | 1.01 | 0.09 |  | 0.88 | 0.77 | - | 1.00 | 0.05 |
|  |  | |  |  |  |  |  |  |  |  |  |  |  |
| TAC of the diet | | |  |  |  |  |  |  |  |  |  |  |  |
|  | Quartiles | |  |  |  |  |  |  |  |  |  |  |  |
|  |  | Q1 | 1 | Ref | | |  |  | 1 | Ref | | |  |
|  |  | Q2 | 1.04 | 0.74 | - | 1.45 |  |  | 1.06 | 0.75 | - | 1.52 |  |
|  |  | Q3 | 0.93 | 0.66 | - | 1.31 |  |  | 0.95 | 0.66 | - | 1.36 |  |
|  |  | Q4 | 1.02 | 0.73 | - | 1.43 |  |  | 1.05 | 0.74 | - | 1.49 |  |
|  | Trend | |  |  |  |  | 0.97 |  |  |  |  |  | 0.99 |
|  | Per 4000 TE/day | | 0.98 | 0.87 | - | 1.10 | 0.76 |  | 0.97 | 0.87 | - | 1.08 | 0.57 |
|  |  | |  |  |  |  |  |  |  |  |  |  |  |

SMC = Swedish Mammography Cohort; COSM = Cohort of Swedish Men; HR = hazard ratio; CI = confidence interval; TAC = Total antioxidant capacity, an index score measured in micromole Trolox equivalents (TE) with the oxygen radical capacity absorbance assay, based on individual answers from FFQ.

^a^ Cox model with attained-age as timescale and restricted to never-smokers, adjusting for intake of alcohol (ethanol, g/day, continuous) and coffee (g/day, continuous), education (compulsory/high school/university), body mass index (<18.5 kg/m^2^, 18.5-24.9 kg/m^2^, 25-29.9 kg/m^2^, ≥30 kg/m^2^), total energy intake (kcal/day, continuous), and multivitamin supplement use (never/sometimes/regular).

**Supplementary Table 4. Associations between daily intake of dietary antioxidants and risk of Parkinson’s disease among ever-smokers**

|  |  | |  | | | | |  |  | | | | |
| --- | --- | --- | --- | --- | --- | --- | --- | --- | --- | --- | --- | --- | --- |
|  |  | | SMC (women) | | | | |  | COSM (men) | | | | |
|  |  | | HR^a^ | 95% CI^a^ | | | *P*-value^a^ |  | HR^a^ | 95% CI^a^ | | | *P*-value^a^ |
| Vitamin C | | |  |  |  |  |  |  |  |  |  |  |  |
|  | Quartiles | |  |  |  |  |  |  |  |  |  |  |  |
|  |  | Q1 | 1 | Ref | | |  |  | 1 | Ref | | |  |
|  |  | Q2 | 1.07 | 0.69 | - | 1.67 |  |  | 1.25 | 0.93 | - | 1.68 |  |
|  |  | Q3 | 0.73 | 0.45 | - | 1.19 |  |  | 1.29 | 0.95 | - | 1.74 |  |
|  |  | Q4 | 0.63 | 0.38 | - | 1.05 |  |  | 1.14 | 0.84 | - | 1.56 |  |
|  | Trend | |  |  |  |  | 0.03 |  |  |  |  |  | 0.77 |
|  | Per 50 mg/day | | 0.86 | 0.73 | - | 1.01 | 0.06 |  | 1.02 | 0.93 | - | 1.12 | 0.65 |
|  |  | |  |  |  |  |  |  |  |  |  |  |  |
| Vitamin E | | |  |  |  |  |  |  |  |  |  |  |  |
|  | Quartiles | |  |  |  |  |  |  |  |  |  |  |  |
|  |  | Q1 | 1 | Ref | | |  |  | 1 | Ref | | |  |
|  |  | Q2 | 0.77 | 0.48 | - | 1.25 |  |  | 1.05 | 0.79 | - | 1.40 |  |
|  |  | Q3 | 0.87 | 0.55 | - | 1.38 |  |  | 1.16 | 0.87 | - | 1.54 |  |
|  |  | Q4 | 0.78 | 0.49 | - | 1.26 |  |  | 0.87 | 0.64 | - | 1.19 |  |
|  | Trend | |  |  |  |  | 0.39 |  |  |  |  |  | 0.84 |
|  | Per 1.2 mg/day | | 0.91 | 0.77 | - | 1.07 | 0.25 |  | 0.95 | 0.88 | - | 1.03 | 0.21 |
|  |  | |  |  |  |  |  |  |  |  |  |  |  |
| ß-carotene | | |  |  |  |  |  |  |  |  |  |  |  |
|  | Quartiles | |  |  |  |  |  |  |  |  |  |  |  |
|  |  | Q1 | 1 | Ref | | |  |  | 1 | Ref | | |  |
|  |  | Q2 | 0.60 | 0.38 | - | 0.94 |  |  | 0.92 | 0.69 | - | 1.22 |  |
|  |  | Q3 | 0.54 | 0.34 | - | 0.87 |  |  | 0.82 | 0.61 | - | 1.10 |  |
|  |  | Q4 | 0.50 | 0.31 | - | 0.81 |  |  | 0.85 | 0.64 | - | 1.14 |  |
|  | Trend | |  |  |  |  | 0.01 |  |  |  |  |  | 0.20 |
|  | Per 2 mg/day | | 0.76 | 0.63 | - | 0.92 | 0.00 |  | 0.95 | 0.85 | - | 1.06 | 0.35 |
|  |  | |  |  |  |  |  |  |  |  |  |  |  |
| TAC of the diet | | |  |  |  |  |  |  |  |  |  |  |  |
|  | Quartiles | |  |  |  |  |  |  |  |  |  |  |  |
|  |  | Q1 | 1 | Ref | | |  |  | 1 | Ref | | |  |
|  |  | Q2 | 0.81 | 0.51 | - | 1.28 |  |  | 0.79 | 0.58 | - | 1.08 |  |
|  |  | Q3 | 0.66 | 0.41 | - | 1.08 |  |  | 0.97 | 0.73 | - | 1.31 |  |
|  |  | Q4 | 0.70 | 0.43 | - | 1.13 |  |  | 0.95 | 0.70 | - | 1.28 |  |
|  | Trend | |  |  |  |  | 0.12 |  |  |  |  |  | 0.71 |
|  | Per 4000 TE/day | | 0.81 | 0.67 | - | 0.98 | 0.03 |  | 1.03 | 0.94 | - | 1.14 | 0.50 |
|  |  | |  |  |  |  |  |  |  |  |  |  |  |

SMC = Swedish Mammography Cohort; COSM = Cohort of Swedish Men; HR = hazard ratio; CI = confidence interval; TAC = Total antioxidant capacity, an index score measured in micromole Trolox equivalents (TE) with the oxygen radical capacity absorbance assay, based on individual answers from FFQ.

^a^ Cox model with attained-age as timescale and restricted to ever-smokers, adjusting for intake of alcohol (ethanol, g/day, continuous) and coffee (g/day, continuous), education (compulsory/high school/university), body mass index (<18.5 kg/m^2^, 18.5-24.9 kg/m^2^, 25-29.9 kg/m^2^, ≥30 kg/m^2^), total energy intake (kcal/day, continuous), and multivitamin supplement use (never/sometimes/regular).
